# Soft Array Surface-Changing Compound Eye

**DOI:** 10.3390/s21248298

**Published:** 2021-12-11

**Authors:** Yu Wu, Chuanshuai Hu, Yingming Dai, Wenkai Huang, Hongquan Li, Yuming Lan

**Affiliations:** 1 Laboratory Center, Guangzhou University, Guangzhou 510006, China; wuyu8320@gzhu.edu.cn; 2 School of Mechanical & Electrical Engineering, Guangzhou University, Guangzhou 510006, China; 1807200159@e.gzhu.edu.cn (C.H.); 1807400027@e.gzhu.edu.cn (Y.D.); 1807300127@e.gzhu.edu.cn (H.L.); 1807200107@e.gzhu.edu.cn (Y.L.)

**Keywords:** artificial compound eye, soft, infrared sensor array, pneumatic

## Abstract

The field-of-view (FOV) of compound eyes is an important index for performance evaluation. Most artificial compound eyes are optical, fabricated by imitating insect compound eyes with a fixed FOV that is difficult to adjust over a wide range. The compound eye is of great significance in the field of tracking high-speed moving objects. However, the tracking ability of a compound eye is often limited by its own FOV size and the reaction speed of the rudder unit matched with the compound eye, so that the compound eye cannot better adapt to tracking high-speed moving objects. Inspired by the eyes of many organisms, we propose a soft-array, surface-changing compound eye (SASCE). Taking soft aerodynamic models (SAM) as the carrier and an infrared sensor as the load, the basic model of the variable structure infrared compound eye (VSICE) is established using an array of infrared sensors on the carrier. The VSICE model is driven by air pressure to change the array surface of the infrared sensor. Then, the spatial position of each sensor and its viewing area are changed and, finally, the FOV of the compound eye is changed. Simultaneously, to validate the theory, we measured the air pressure, spatial sensor position, and the FOV of the compound eye. When compared with the current compound eye, the proposed one has a wider adjustable FOV.

## 1. Introduction

Compared with the single aperture eyes of vertebrates, insect compound eyes have the characteristics of a wide field of view (FOV) [1,2]. How to better tap the value of artificial compound eyes has recently become a hot research topic [3,4]. Improving the adjustable range of the FOV of compound eyes is the key to developing compound eyes that are adaptable to complex and extreme working environments. In some complex and extreme environments, compound eyes need to have a wider adjustable FOV, such as: when tracking from multiple objects to one object, or from one object to multiple objects; when tracking high-speed objects (to make up for the deficiency that high-speed objects cannot be fully tracked due to the rudder unit matched with the compound eye or the slow response speed with the steering platform). Therefore, by improving the adjustable range of the compound eye’s FOV, it can be adapted to more complex and extreme working environments, and the application space of the compound eye can be considerably improved.

**Figure 1:**
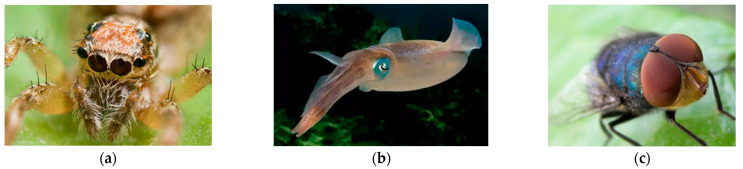
(**a**) The eight-eyed jumping spider with an annular compound eye structure has a 360° FOV; (**b**) Squid eyes with the ability to change their FOV. (**c**) Insect compound eye capable of detecting high-speed objects.

Most artificial compound eyes have a multilayer optical lens; the manufacturing process of the planar compound eye is simple, but its FOV is narrow. A curved compound eye has a wide FOV, but its manufacturing process is complex [1,3,5,6]. As shown in Figure 1, to combine the advantages of the planar compound eye and curved compound eye, this study combines the characteristics of the annular compound eye of the eight-eyed jumping spider with a wide FOV [7], a squid eye with a variable FOV [8], and the insect compound eye with high-speed detection ability [9]. The proposed soft-array surface-changing compound eye (SASCE) is as follows: a high-performance bionic compound eye, which can change its FOV. The sensors are attached to the upper surface of the soft pneumatic model to form an array, and the upper surface of the soft pneumatic model is driven to bulge through the air pressure difference. The array surface changes with deformation of the soft pneumatic model, which leads to a change in the spatial position and viewing area of each sensor, and finally changes the FOV of the compound eye (SASCE). The change results of each sensor are closely related to its initial position, the thickness distribution of the soft aerodynamic model (SAM), and air pressure. Through multiple groups of experimental measurements of the SAM, a more accurate relationship can be derived between the theoretical FOV of the compound eye and the air pressure. The SASCE proposed here can change the FOV according to the requirements, to allow it to adapt to the environmental and working requirements. The innovations of this paper are as follows: (1) a wide application range: the size of the FOV can be changed by pneumatically driving the compound eye model, meaning that it can be applied to more working environments; (2) the ability to protect precision components on the carrier: the carrier is made of silicone rubber, which can provide a buffer and better protect valuable components such as sensors to a certain extent; (3) it is combined with the design of bionics: inspired by various biological species with special eye structures, a high-performance bionic compound eye with a large FOV is obtained using the characteristics of the annular compound eye of the eight-eyed jumping spider, squid eye with a variable FOV, and insect compound eye with a high-speed detection ability.

## 2. Structural Design and Principle

The designed SASCE has two different states—flat compound eye and curved compound eye—and the SASCE has different advantages in different states. The SASCE has a wider FOV when it is curved. With a visual sensor as the load, the overlapping area of the SASCE can make the view clearer in both flat and curved compound eyes at a low pressure [5]. With infrared sensing as the load, the heat source in the overlapping area, as seen by SASCE, can be more accurately located in space [10]. In this study, a software array driven by air pressure difference is established to change the compound eye, so that the FOV of the compound eye can be adjusted over a wide range. Here, the variable structure infrared compound eye (VSICE) comprising seven infrared array sensors is used to illustrate and verify this method. Thus, a compound eye with a wider adjustable field angle can be developed. The SASCE uses air pressure to drive the load surface to expand, to change the array surface of the sensor and, finally, change the FOV. To control this transformation and improve the manufacturing efficiency, this study adopted the manufacturing method of integrated multiple-film inversion forming. To ensure the air tightness of the pneumatic components of the compound eye and make the components replaceable, the double-layer structure design can be adopted to control the expansion and reduction of the compound eye by driving the airbag liner through the air-pressure difference. The outer membrane is used to attach the sensor to change the FOV of the compound eye.

**Figure 2:**
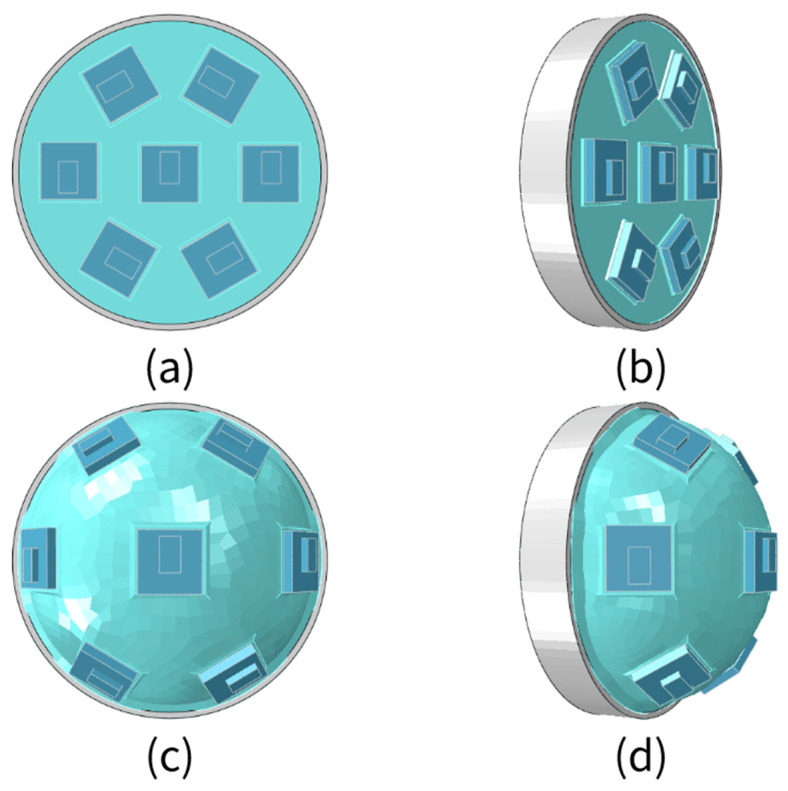
The soft array surface changes the basic structure of the compound eye. (**a**) Top view of unfilled gas; (**b**) Oblique view of unfilled gas; (**c**) Top view of filled gas; (**d**) Oblique view of filled gas.

First, the SASCE is shown in Figure 2. The sensor array is distributed on the upper surface of the SAM in a regular hexagonal rotation arrangement. When gas is not filled, the infrared sensor array surface is flat, as shown in Figure 2a,b. As gas is filled, the compound eye is driven to expand through the internal and external air-pressure difference, the spatial position of the sensor and the change in viewing area, and the infrared sensor array surface becomes curved, as shown in Figure 2c,d. At this time, the FOV of the compound eye becomes larger. The SASCE is driven by the internal and external air pressure difference. It can increase the FOV of the compound eye by increasing the internal and external air pressure difference, and reduce the FOV of the compound eye by reducing the internal and external air pressure difference, creating a wide-ranging, changing FOV. By collecting and analyzing data from the sensors on the compound eye under different internal and external air-pressure differences, we can verify the accuracy and feasibility of the theoretical method proposed here.

**Figure 3:**
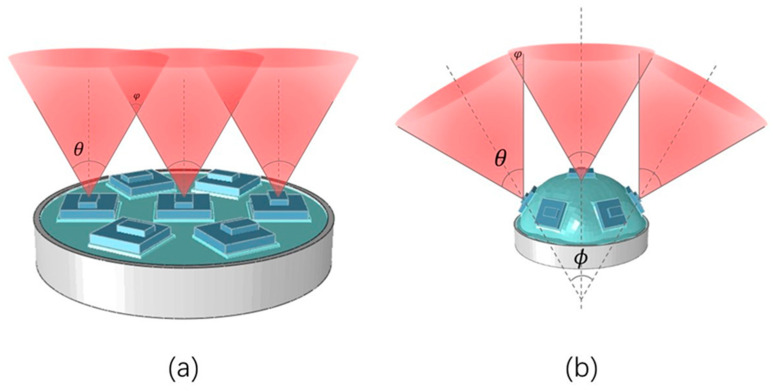
(**a**) SASCE is the FOV of the plane; (**b**) SASCE is the FOV of the surface.

The FOV of SASCE has an important relationship with the SAM state. The FOV of the compound eye is λ; the FOV of the sensor is θ; the intersection angle of the FOV of the center sensor; the side sensor is φ. As shown in Figure 3a, when the SASCE is not filled with gas, the FOV of the compound eye can be regarded as the FOV of the sensor, i.e., λ = θ. According to the geometric relationship, φ = θ. As shown in Figure 3b, when the SASCE is filled with gas, the surface of the software array changes. The visual-field center of the compound eye becomes consistent with the sensor at the center point. The upper surface of each sensor is parallel to the SAM. The visual-field centerline of the sensors on both sides intersects with the visual-field centerline of the central sensor at one point. At this point, the intersection of the visual-field centerline of the sensors on both sides is the included angle enlarge ϕ, which includes angle changes with the change in the compound eye sensor position under different increases in air pressure. According to the geometric relationship, the relationship between the FOV of SASCE and the FOV of the sensor is given as

(1) λ=θ+ϕ

The relationship between the intersection angle of the FOV of the central sensor and the side sensor and the FOV of SASCE is as follows:

(2) $$\varphi =\theta -\frac{1}{2}\phi$$

Controlling the change in the air pressure can change the FOV of compound eye.

This paper only uses the above principles and infrared sensors to verify this. On this basis, other, more efficient sensors or cameras can be used, and different array methods can be adopted for further design.

## 3. Model Analysis

### 3.1. Structural Analysis of SAM

For a structural analysis of the model, the SAM was simplified as a large deflection problem of a circular thin plate. This study provides the following two ways of establishing a mathematical model for the problem.

#### 3.1.1. The Problem Is Solved by the Von Karman Equation

The problem of the large deflection of a thin circular plate under uniformly distributed pressure can be treated using the von Karman equation [11], which can be written in the dimensionless form as follows:

(3) $$S_{t}=S_{r}+2x\frac{{\mathrm{dS}}_{r}}{\mathrm{dx}}$$

(4) $$\frac{d^{2}}{{\mathrm{dx}}^{2}}\left({\mathrm{xS}}_{r}\right)+\frac{1}{2}{\left(\frac{\mathrm{dw}}{\mathrm{dx}}\right)}^{2}=0$$

(5) $$\frac{3}{16}P+\frac{3}{4}\left(1-v^{2}\right)S_{r}\frac{\mathrm{dw}}{\mathrm{dx}}-\frac{1}{4}\frac{d^{2}}{{\mathrm{dx}}^{2}}\left(x\frac{\mathrm{dw}}{\mathrm{dx}}\right)=0$$

In Equations (3)–(5), the immeasurable stiffness $W\left(x\right)=\frac{w}{h}$, $P=\frac{a^{4}q}{h^{4}E}\left(1-v^{2}\right)$, $S_{r}\left(x\right)=\frac{a^{2}}{{\mathrm{Eh}}^{3}}N_{r}$, $S_{t}\left(x\right)=\frac{a^{2}}{{\mathrm{Eh}}^{3}}N_{t}$.

The immeasurable stiffness coordinate x is defined as: $x=\frac{r^{2}}{a^{2}}$, 0≤x≤1, where h is the plate thickness, a is the plate radius, q is the uniform load density, E is the Young’s modulus, v is the Poisson’s ratio, r is the radial coordinate, $N_{r}$ is radial membrane force, $N_{t}$ is the tangential membrane force, and w is the normal phase shift of the plate at distance r from the center of the plate.

Ji-Huan He studied the problem of large deflection in a circular thin plate, and proposed an approximate solution to the maximum deflection at the center point as follows:

(6) $$\frac{3}{16}P=W_{m}+{\beta W}_{m}^{3}$$

(7) $$\beta =\frac{3\left(1-v^{2}\right){\left(5C-2\right)}^{2}}{200\left(3C^{2}+4C+4\right)}$$

(8) $$C=-\frac{3-v}{1-v}$$

(9) $$P=\frac{a^{4}q}{h^{4}E}\left(1-v^{2}\right)$$

In Equations (6)–(9), $W_{m}$ = W(0), v is the Poisson’s ratio, a is the plate radius, h is the plate thickness, q is the pressure, and E is the Young’s modulus.

#### 3.1.2. Solving Large Deflection with Governing Equation

The governing equation of the axisymmetric large section bending of a circular thin plate [12] is given as follows:

(10) $$D\frac{d}{\mathrm{dr}}\left(\nabla^{2}w\right)=\varphi +\frac{h}{r}\frac{d\emptyset }{\mathrm{dr}}\frac{\mathrm{dw}}{\mathrm{dr}}\;\frac{d}{\mathrm{dr}}\left(\nabla^{2}\emptyset \right)=-\frac{E}{2r}{\left(\frac{\mathrm{dw}}{\mathrm{dr}}\right)}^{2}$$

In Equation (10), w is the deflection of the plate, ∅ is the membrane stress function, h is the thickness, r is the radial coordinate, $\nabla^{2}$ is a numeric operator,

(11) $$\nabla^{2}\left(\cdots \right)=\frac{1}{r}\frac{d}{dr}\left[r\frac{d\left(\cdots \right)}{dr}\right]$$

φ is a load function, $\varphi =\frac{1}{r}\int_{0}^{r}qrdr$; D is the statistical characteristic of the plate,

(12) $$D=\frac{Eh^{3}}{12\left(1-\mu^{2}\right)}$$

E is the Young’s modulus, and μ is the Poisson’s ratio. The boundary conditions of the governing equation are:

(13) $$r=0,\frac{\mathrm{dw}}{\mathrm{dr}}=0,\sigma_{r}\;\mathrm{is}\;\mathrm{limited},\;\text{r}=\text{a},\text{w}=0,{\text{M}}_{\text{r}}=0,{\text{σ}}_{\text{r}}=0$$

In Equation (13), $\sigma_{r}$ represents radial stress and $M_{r}$ is the bending moment.

(14) $$\sigma_{r}=\frac{1}{r}\frac{d\emptyset }{\mathrm{dr}}$$

(15) $$M_{r}=-D\left(\frac{d^{2}w}{{\mathrm{dr}}^{2}}+\frac{\mu }{r}\frac{\mathrm{dw}}{\mathrm{dr}}\right)$$

The above two mathematical models can be solved by methods such as iteration [13,14], variational principle [15], Ritz method [16], and semi-inverse solution [17], and approximate solutions to the upper-surface displacement of circular thin plates at different positions can be obtained.

### 3.2. SAM Finite Element Analysis

**Figure 4:**
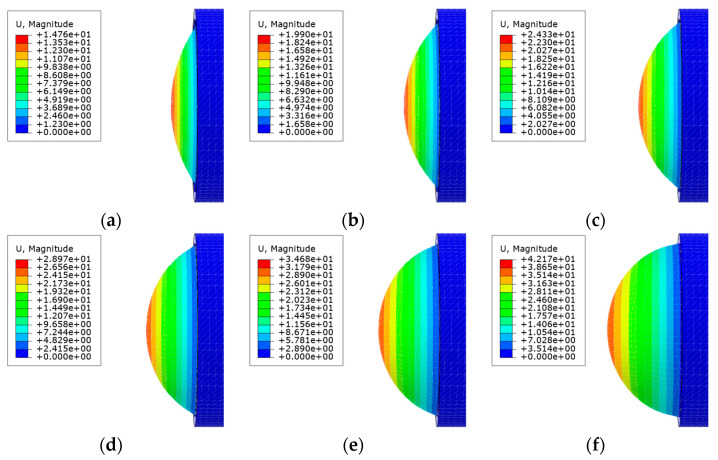
Based on the ABAQUS software, the model state renderings of the CTPM under different air pressures are simulated. (**a**–**f**) is the simulation effect diagram of CTPM from the relative ambient air pressure 6 kPa–36 kPa with a gradient of 6 kPa.

**Figure 5:**
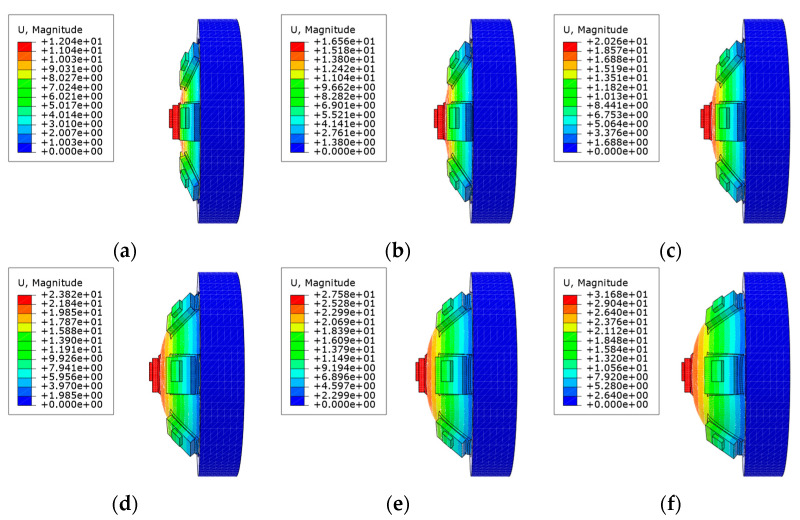
The ABAQUS software simulates the model state effect diagram of SAM under different air pressures. (**a**–**f**) is the simulation effect diagram of the SAM from relative ambient air pressure 6 kPa–36 kPa with a gradient of 6 kPa.

The relationship between the stress and strain of the silica gel material was obtained by a tensile test. The strain potential energy density function is the best effect of Yeoh’s third-order parameter model. A hyperelastic model of silica gel material Yeoh3 was pbtained, in which C10 = 90036 Pa, C20 = −3880.6 Pa, C30 = 1524 Pa, and density $\rho =1072.7\mathrm{kg}/m^{3}$ [18]. As the materials used are hyperelastic, predicting the large deformation of a circular thin plate model (CTPM) and SAM is challenging. Therefore, we used finite element analysis (FEA) to intuitively analyze the deformation of a circular thin plate and soft aerodynamic compound eye SAM, and took its expansion process as the evaluation standard. Based on the software, ABAQUS, the influence of air pressure on the deformation of CTPM and SAM with the same thickness was simulated, and the model state diagrams under different air pressures were obtained, as shown in Figure 4 and Figure 5.

**Figure 6:**
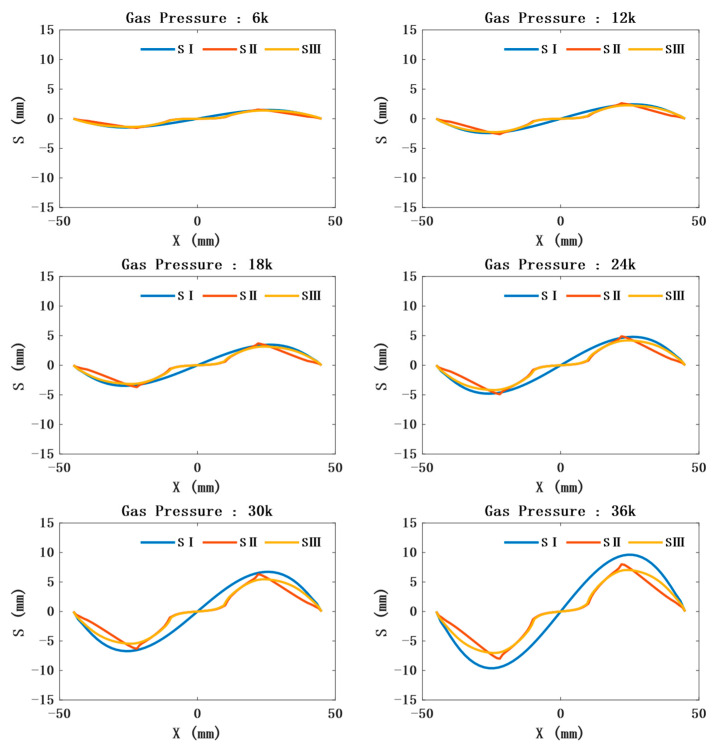
Relationship between the distance from the central axis of the model (the horizontal position relative to the ambient air pressure of 0 Pa) and the horizontal displacement under different air pressures. SI represents the relationship between the distance of the CTPM from the central axis and displacement under different air pressures. SII represents the relationship between SAM’s maximum constraint, that is, the distance between each point on the line constrained by three sensors and passing through the central axis, and the horizontal displacement. SIII represents the relationship between SAM’s minimum constraint, that is, the distance between each point on the line constrained by only one sensor in the center and passing through the central axis, and the horizontal displacement.

The large deflection of circular thin plates is axisymmetric, and a line passing through the center point of its upper surface can be used to replace the movement of the whole expansion surface; SAM is designed based on CTPM. Through data acquisition and analysis of lines with the most constraints (minimum shape variable) passing through the central axis, and the lines with the least constraints (maximum shape variable) passing through the neutral axis, the change law of the model with air pressure can be obtained. By comparing this with CTPM, the change law of SAM and CTPM with air pressure can be obtained under the condition of seven sensor arrays. Through analysis, we can obtain the following results:

By predicting the horizontal displacement of the model, the horizontal position of the sensor under different air pressures can roughly be determined. The direction of the air pressure force is always perpendicular to the surface of the stressed object; thus, the displacement in the horizontal direction of a CTPM point first increases and then decreases with the increase in the horizontal distance from the center point, and the displacement also increases with the increase in air pressure, as shown in the curve Si of Figure 6. As SAM is constrained by the sensor relative to CTPM, its horizontal displacement becomes smaller and more obvious with an increase in air pressure. In the local range, the displacement law without the sensor constraint is roughly the same as that of CTPM, and the displacement law of the sensor constraint is approximately linear.

**Figure 7:**
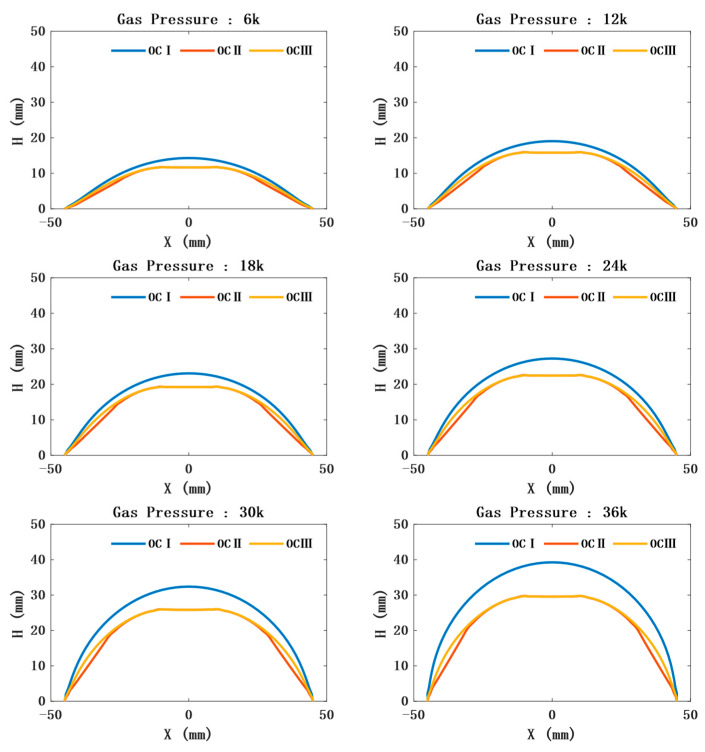
Outline drawing under different air pressures. OCI represents the outline of CTPM under different air pressures; OCII represents the contour of the line where SAM is constrained the most, that is, it is constrained by three sensors and passing through the central axis; OCIII represents the contour of the line whose SAM is constrained the least, that is, it is constrained by only one sensor in the center and passing through the central axis.

**Figure 8:**
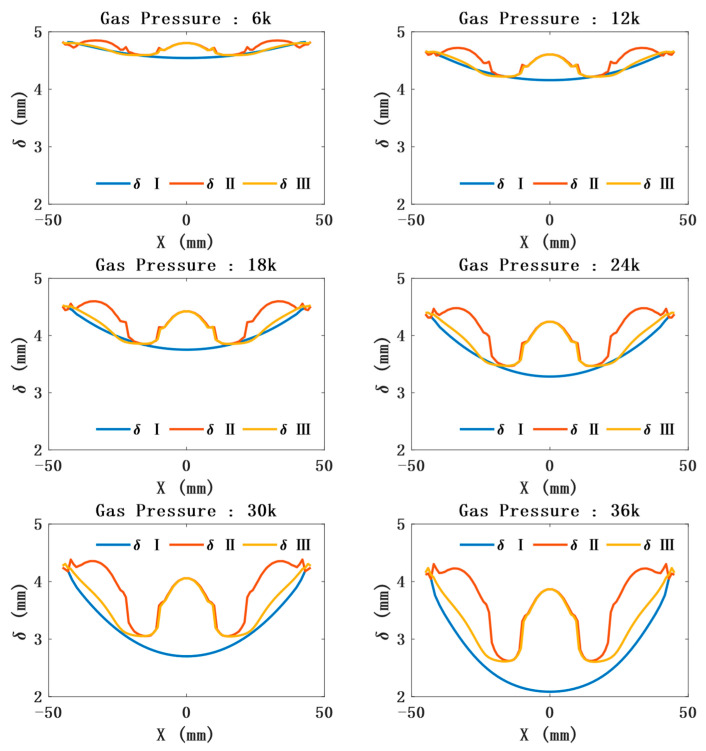
Relationship between the distance from the central axis (the distance relative to the ambient air pressure of 0 Pa) and the thickness change under different air pressures. δI represents the thickness variation law of a circular thin plate; δII represents the thickness variation law of the soft pneumatic compound eye model with the maximum constraint; that is, it is constrained by three sensors and passing through the central axis; δIII represents the thickness variation law of the soft pneumatic compound eye model with the least constraint; that is, it is constrained by only one sensor, located in the center and passing through the central axis.

1. The position and orientation of the sensor can be further obtained by predicting the outer contour of the model. In the limited air-pressure range, the outer contour of CTPM tends to be spherical under the action of air pressure. The radius of the ball decreases and the center angle increases with an increase in air pressure. Due to the sensor constraints, the contour of the unconstrained part of the CAM is approximately spherical, and its radius and spherical center angle are consistent with the variation law of the radius and spherical center angle of a circular thin plate with a change in air pressure. However, its contour radius is greater than the contour radius of CTPM under the same air pressure, and the spherical center angle is less than the spherical center angle of CTPM under the same air pressure. The contour of the constrained part is approximately plane, as shown in Figure 7.
2. The prediction of the model thickness is important for the safety of SAM. CTPM expands under the action of air pressure, while its plate thickness decreases with an increase in air pressure, and the closer it is to the central axis, the thinner it becomes. The thickness of SAM at the constrained position will first increase and then decrease. However, with an increase in air pressure, the overall thickness still shows a thinning trend, and the thickness at the unconstrained part is close to the thickness of CTPM, as shown in Figure 8.

## 4. Fabrication

### 4.1. Production of SAM

Most software robots are made by making two halves of the robot and then using adhesive to stick the two halves together. This method makes the robot easy to break when charged with high air pressure, which greatly reduces the service life of the robot. If new silica gel fluid and incompletely solidified silica gel are used to penetrate and bond each other [19], fabricating the robot becomes complex. Combined with the requirements of the compound eye, this study adopted the method of integrated forming. The manufacturing process of the compound eye is as follows:

Step 1: We chose medical silica gel (PS6600, Shenzhen Yipingyiping Model Material, China) and mixed silica gel A and B in equal amounts, stirring with a glass rod for ~1 min. After they were evenly mixed, they were placed in a vacuum barrel to extract air by vacuum, and then the mixture was left to sit for another 5 min.

**Figure 9:**
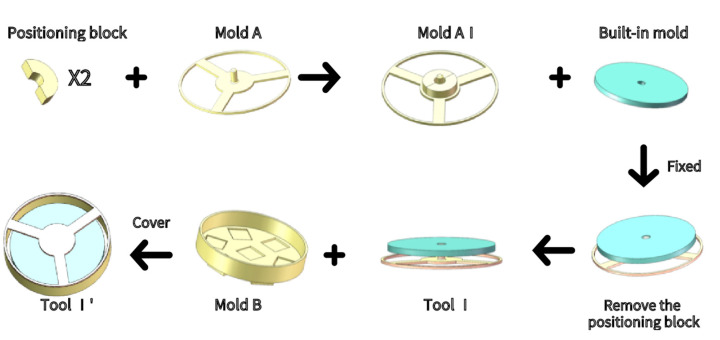
SAM mold preparation.

Step 2: As shown in Figure 9, we combined two semi-circular Positioning blocks and placed them on Mold A to form Mold AI. The built-in mold was tightly fastened on Mold AI and fixed with a hot-melt adhesive. The positioning block was removed to obtain the combined abrasive Tool I. The combined abrasive Tool I was tightly buckled on the Mold B and fixed and sealed with hot-melt adhesive to obtain the combined abrasive Tool I’.

**Figure 10:**
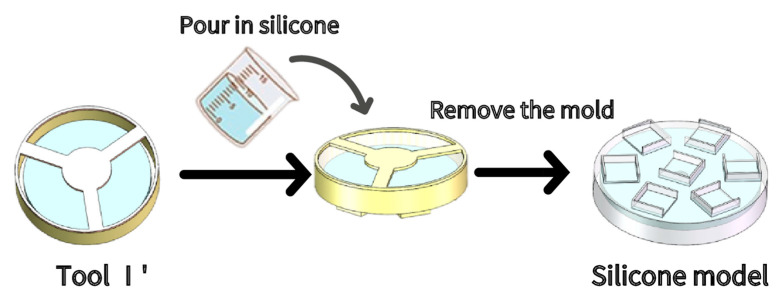
SAM main body production.

Step 3: As shown in Figure 10, we slowly poured the newly prepared silica gel solution into Tool I’, and then placed it into the vacuum barrel. The vacuum machine was used to extract air in the vacuum barrel. After waiting for ~5 min, it was taken out, and the newly prepared silica gel solution was poured to fill the Tool I’. The abrasive tool was removed after it solidified, and the built-in mold was left in the Silicone model.

**Figure 11:**
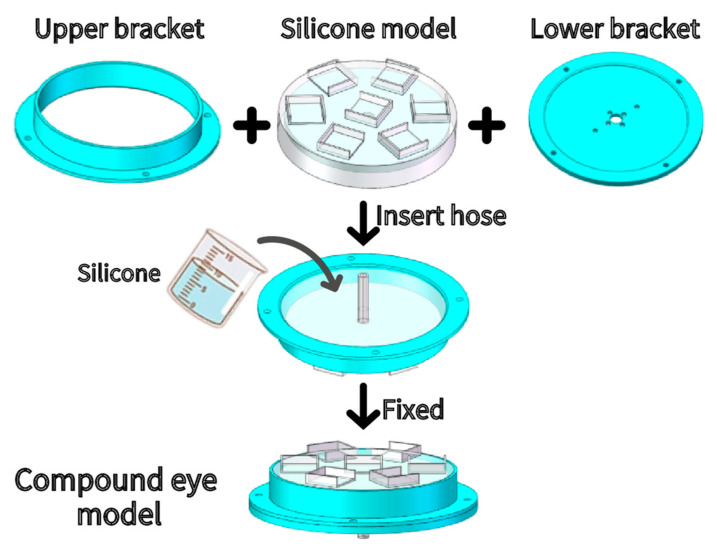
Sam group transfer.

Step 4: As shown in Figure 11, the upper bracket was combined with the model; the rubber hose was inserted, and the newly prepared silica gel solution was poured until it was flat with the edge of the frame. After the silica gel solution solidified, the lower bracket and upper brackets were fixed together through threaded fasteners to obtain the preliminary SAM or Compound Eye model.

**Figure 12:**
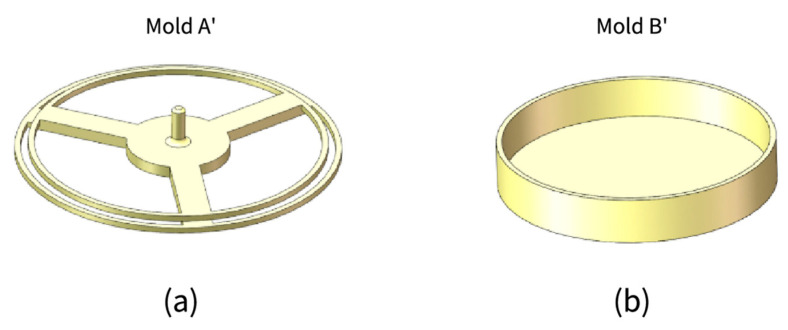
Double-layer structure design and manufacturing of the mold and CTPM manufacture mold. (**a**) Design mold for double-layer structure; (**b**) Mold for CTPM.

The production process of CTPM is similar to that of SAM, except Mold B was replaced with Mold B’ (as shown in Figure 12b). If the double-layer structure design (inner tank and outer membrane) is adopted, and Mold A needs to be replaced with Mold A’ (as shown in Figure 12a), then CTPM is made first, Mold A’ is retained when removing the mold, and the film can be inverted again by Mold B (as shown in Figure 10).

### 4.2. Design of the VSICE Prototype System

**Figure 13:**
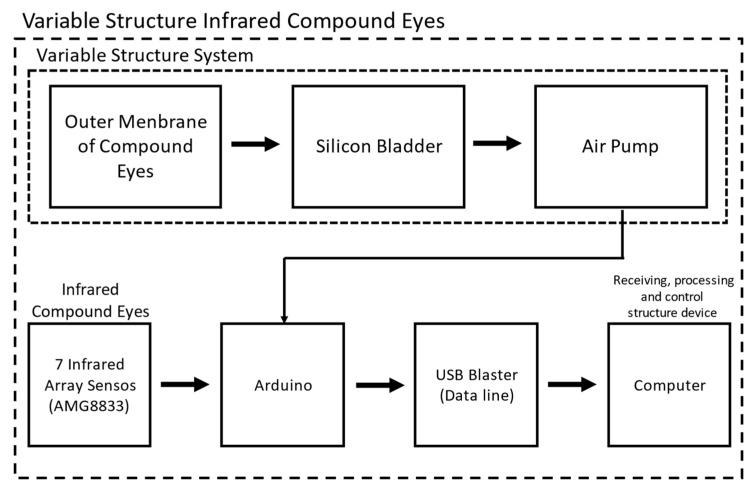
Architecture of the VSICE system: seven infrared array sensors are attached to the SAM, the outer membrane controls the internal air pressure of the SAM through an air pump to change the array surface, and the infrared array sensors are directly connected to Arduino. Data are transmitted in real time through IIC to the computer for processing.

The design of the VSICE prototype system is shown in Figure 13. The VSICE system is mainly composed of three parts—SAM, infrared array and control—and the receiving and processing device. To change the array surface and adjust the FOV angle, the sensor is attached to the outer surface of the SAM, and the expansion of and reduction in the compound eye are controlled using a pneumatic to adjust the FOV.

To realize infrared thermal imaging of the compound eye, we used an 8 × 8 infrared array sensor (model: AMG 8833) as the monocular of the compound eye, and the whole compound eye system was composed of seven sensors. The field of vision of the sensor was 60° up, down, left, and right. The optical center gap of each pixel angle sensor was within ± 5.6° (horizontal and vertical directions). All sensors were connected and controlled through an Arduino development board. The temperature data were transmitted back to the development board through IIC, and the development board transmitted the data to the computer for processing.

### 4.3. Overall Model

**Figure 14:**
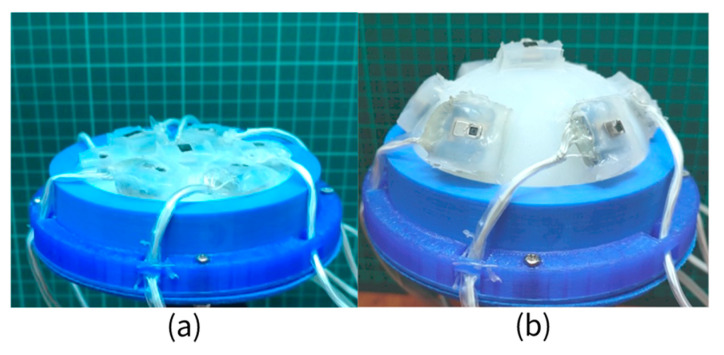
VSICE model. (**a**) The VSICE in the initial state is in the plane compound eye mode. (**b**) The shape of the VSICE in the inflated state is a curved compound eye mode.

As shown in Figure 14a, silica gel was used to install the infrared sensor on the preliminary SAM model, and relevant accessories were installed to obtain a complete VSICE (with the ability to read the heat source and the same mathematical law as SAM). When air is pumped to fill the VSICE with gas, the load surface of the sensor expands, and then the spatial position of each sensor changes regularly, that is, the array surface changes regularly (Figure 14b).

## 5. Experiments and Results

A prototype of the VSICE was developed. To verify the correctness of CTPM and SAM FEA, the applicability of the circular thin plate mathematical theory and the ability of VSICE to change the FOV, the model deformation test experiment and sensor FOV experiment were designed.

### 5.1. Model Deformation Test

**Figure 15:**
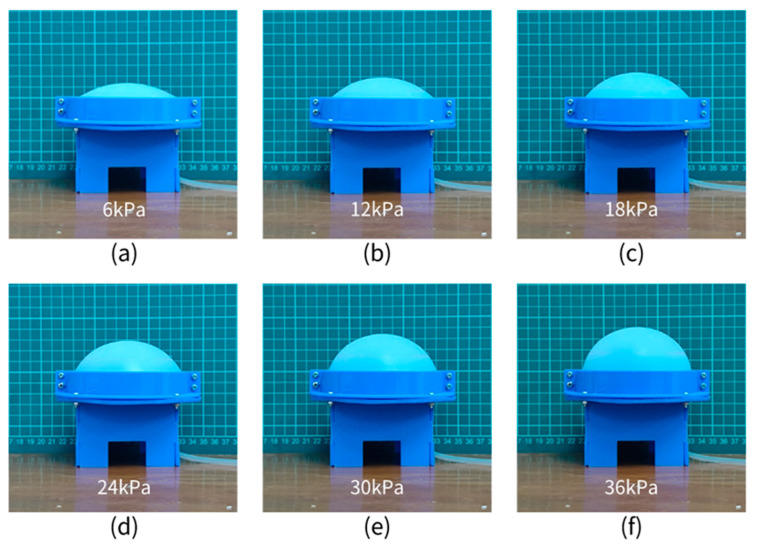
CTPM experimental diagram. (**a**–**f**) respectively represent the state record diagram of the circular thin plate model with 6 kPA–36 kPa pressure increasing at an interval of 6 kPa.

**Figure 16:**
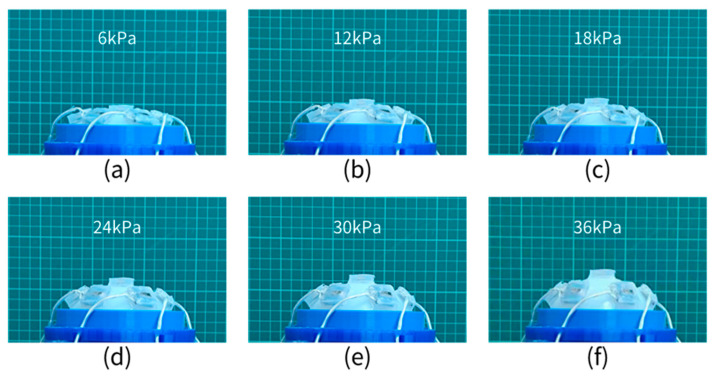
SAM experimental diagram, (**a**–**f**) represent the state recording diagram of the soft pneumatic compound eye model at 6 kPa–36 kPa increasing at an interval of 6 kPa.

To test the reliability of the analysis results of CTPM and SAM, the position where sensors are added on the upper surface of the circular thin plate model were marked, gas was periodically filled into the CTPM and VSICE (with the same mathematical law of air-pressure driving as SAM, which can be regarded as SAM during the model deformation test), and their states were recorded using a camera (as shown in Figure 15 and Figure 16).

The mini air pump with rated voltage of 12 V is used to continuously inflate CTPM, and it takes only 9 s to raise the internal air pressure of CTPM to 36 kPa. By directly comparing the images in Figure 15a–f, we find that, with an increase in air pressure, the outer contour of the CPTM will gradually approach the sphere and the sphere radius will gradually decrease, which preliminarily proves the correctness of the simulation conclusion. According to the partial processing of the image, we can obtain the discrete relationship between the specific outer contour of CTPM, the central point, and the corresponding point of the peripheral sensor placement with changes in air pressure.

The SAM is continuously inflated with a mini air pump with a rated voltage of 12 V, and it takes only 12 s to raise the internal air pressure of the SAM to 36 kPa. By directly comparing the images of Figure 16a–f, we see that the increase law is consistent with the increase law obtained from the CTPM experiment. Compared with the CTPM experiment records, the outer contour radius of the SAM is greater than that of the CTPM under the same air pressure, which further proves the correctness of the simulation conclusion. By collecting the position of the central point at the bottom of the sensor facing the image, the discrete point relationship between the air pressure and the point position can be obtained.

**Figure 17:**
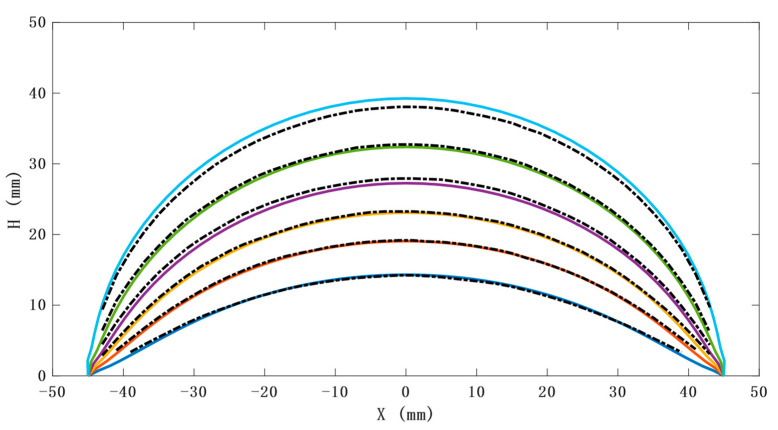
FEA and actual contour of the CTPM under different various air pressure values. The solid line represents the results of FEA based on ABAQUS, and the dotted line represents the actual measurement results.

As the expansion problem of the CTPM under the action of air pressure is an axisymmetric problem, a line contour on the upper surface passing through the central axis of the model can be used to represent the whole model state; then, the outer contour boundary of CTPM images under different air pressures (as shown in Figure 15) is sampled, and the results are compared with the contour results of FEA. As shown in Figure 17, when the air pressure is low, the two contours are almost consistent. With an increase in air pressure, compared with the contour of FEA, the experimental contour has a certain error and a certain fluctuation, which is mainly due to the combination of the error of air pressure sensor and the error of the model material properties during measurement. The contour of the CTPM FEA is consistent with the contour of the CTPM experiment.

**Figure 18:**
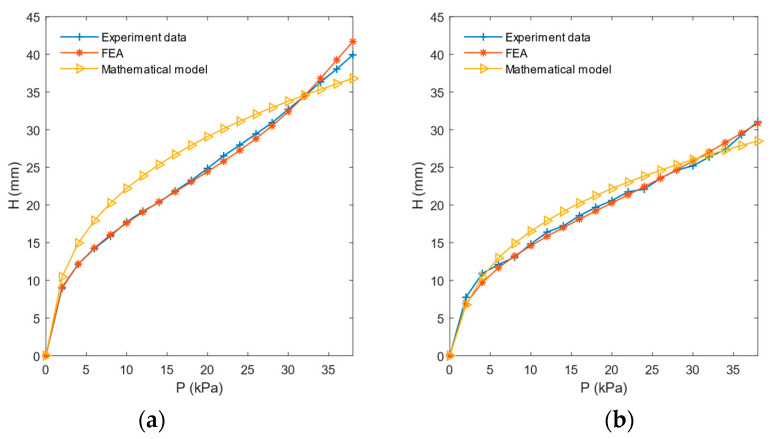
Relationship between CTPM and SAM center point displacement and air pressure. (**a**) Relationship between CTPM center point displacement and air pressure and (**b**) relationship between SAM center point displacement and air pressure. Experimental data represent the relationship between center point displacement measured by the model and air pressure. FEA represents the relationship between center point displacement and air pressure based on the ABAQUS finite-element analysis. Mathematical model, which models data into Equations (5)–(8), represents the relationship between the displacement of the center point and the air pressure.

By increasing the sampling frequency of collecting air pressure for CTPM and SAM, the center point displacement of the collected results is derived. As shown in Figure 18, the curve of the FEA result of the model is almost consistent with the measured result curve, and there is a large gap between the measured results of the mathematical model and CTPM, because Equations (5)–(8) are approximate solutions of circular thin plates, and not accurate solutions. The mathematical model corrected by the elastic modulus of SAM is close to the actual results.

**Figure 19:**
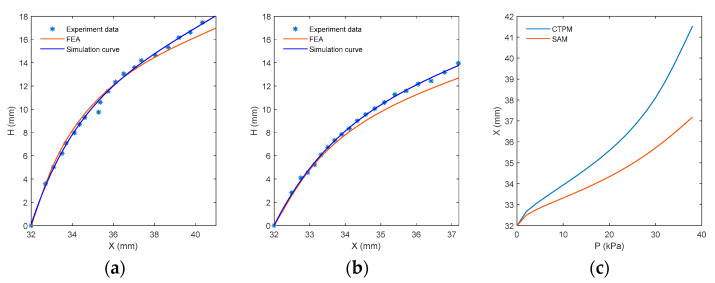
Horizontal distance–vertical displacement and air pressure–horizontal distance diagrams of the outer ring points (attachment point of SAM’s outer ring sensor and the corresponding point of CTPM’s outer ring sensor attachment point). (**a**,**b**) represent the outer ring point displacement path curves of CTPM and SAM, respectively, where experimental data represent the measured point displacement results, and FEA represents the simulated point displacement path curve. The simulation curve represents the curve fitted according to the actual measurement points. (**c**) represents the pressure level position relationship curve obtained from CTPM and SAM finite-element analysis.

The deformation of SAM can be approximately regarded as an axisymmetric problem. The point displacement path of one outer ring sensor can be used to take the paths of all outer ring sensors representing SAM, change the sampling density, and derive the moving paths of outer ring points of SAM and CTPM. As shown in Figure 19, the displacement road force of FEA results is close to the actual displacement path, but there is still a certain error. This is mainly caused by a certain amount of silica gel spilling and adhering to the upper surface of the model when installing the sensor, resulting in the local thickness of the actual model not being completely consistent with the FEA model. The tangential direction of the path curve is the sensor direction, which can be combined with the air-pressure horizontal distance relationship curve to obtain the approximate displacement of the sensor under different air pressures and the area shown by the sensor.

#### VSICE FOV Experiment

**Figure 20:**
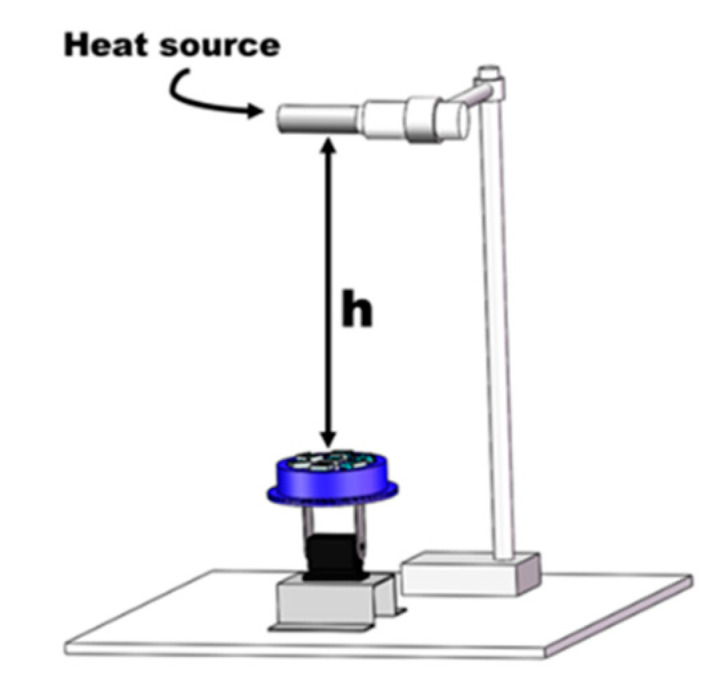
Schematic diagram of the test VSICE experimental platform.

To verify the ability of VSICE to control the change of FOV, the performance of the compound eye under different air pressures was verified. A schematic diagram of the experimental test is shown in Figure 20; in the experiment, the VSICE is controlled by the steering gear pan tilt, the air pressure sensor is used to read the air pressure data, and the infrared sensor is used to read the data of the heat source. By adding a heat source in each sensor, the repetition range of the FOV size and the FOV area can be judged. The visual-field performance of the compound eye under the corresponding air pressure was tested.

**Figure 21:**
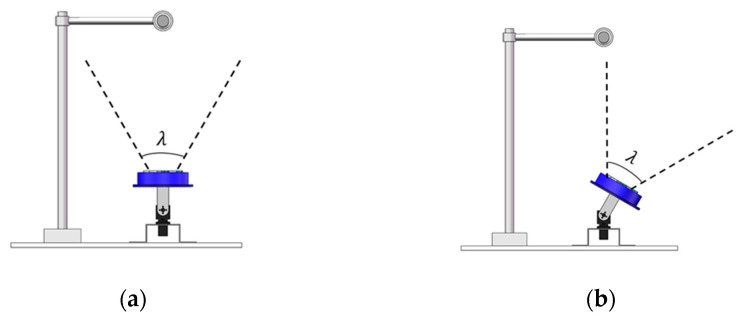
Schematic diagram of the FOV experiment. (**a**) The steering gear pan tilt was used to level the center sensor. (**b**) Using a rotation angle of $\varphi_{i}$ for the steering gear pan tilt, the heat source was imaged at the edge of the FOV.

The data of the heat source are read at a height of h = 250 mm, directly above the compound eye. When the compound eye is in the horizontal position, the compound eye can read the infrared array data in the front view. At this time, the steering gear pan tilt below the compound eye controls its vertical rotation (Figure 21). When the heat source image of the side eyelet of the compound eye is at the edge of the array, the rotation angle of the steering gear is $\varphi_{i}$, half the field angle of the compound eye; then, the field angle of the compound eye under the corresponding air pressure is $\varphi =2\varphi_{i}$.

**Figure 22:**
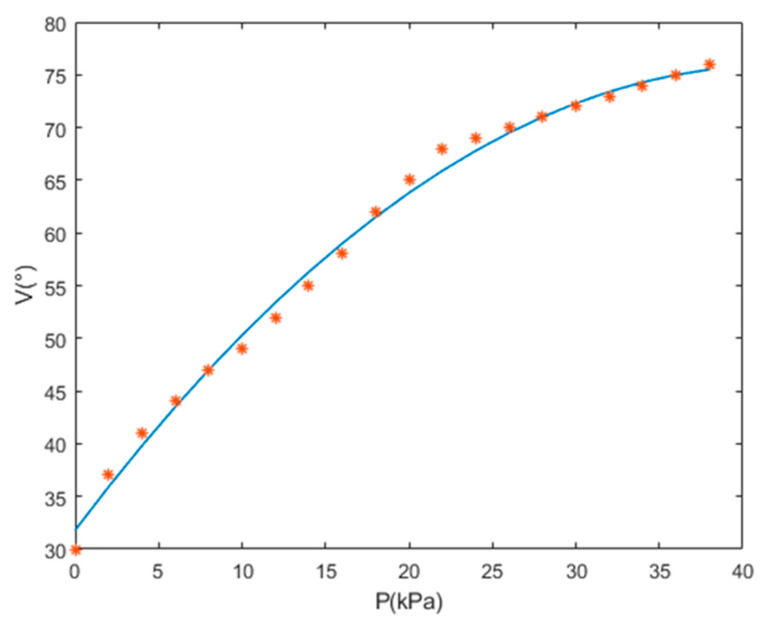
VSICE FOV curve, where V is half the FOV of VSICE.

Through the FOV experiment of VSICE under 0–38 kPa, the relationship curve between air pressure and half of the FOV of compound eye can be obtained (Figure 22). This curve can be reflected in the corresponding performance of the sensor FOV under different air pressures. VSICE has a good performance in FOV expansion. When the maximum air pressure is 38 kPa, the FOV of the compound eye can reach 150°.

Through the actual FOV test of the compound eye, it can be fitted into a FOV field control curve regarding the air pressure P. The curve shows that when the air pressure of the compound eye is 0, the FOV of the compound eye is the original FOV of the sensor. When the compound eye is inflated, the FOV of the compound eye can quickly reach 140° in the process of low air-pressure changes. When the air pressure exceeds 20 kPa, the FOV of the compound eye gently changes. The FOV of the compound eye reached 150° until the maximum test pressure was 38 kPa. By fitting the corresponding air pressure P with the corresponding field angle V, the relationship became close to a quadratic polynomial curve, that is, $V=k_{1}p^{2}+k_{2}p+C$ (where $k_{1},k_{2},\mathrm{and}\;C$ are the coefficient of the quadratic multinomial). The value of the coefficient is affected by factors such as sensor array position and material properties. Under this prototype, the corresponding value is $k_{1}=-0.025;\;k_{2}=2.1;\;C=31.8$, and the FOV of a soft-body pneumatic compound eye λ=2V. From this relationship, the corresponding field angle can be obtained by controlling the air pressure.

Taking the array data read by sensors at pressure of 0 kPa, 18 kPa, and 36 kPa as an example, the data are processed and converted into thermographic images, which show the different performances of each small eye when the compound eye is tested with a single heat source under different pressures.

**Figure 23:**
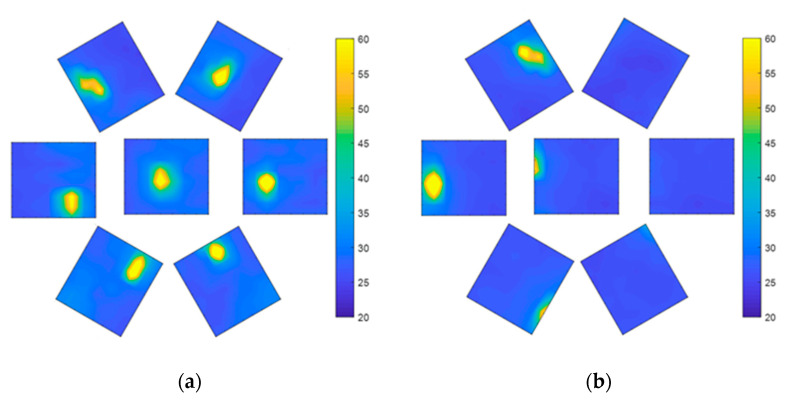
FOV test at 0 Pa; (**a**) Compound eye front test image. (**b**) An image in which the compound eye is rotated to the edge of the FOV.

When the air pressure is 0 kPa, VSICE can be regarded as a plane array compound eye, and the seven sensors can measure the heat source, as shown in Figure 23a. After the steering gear rotates by 25°, the position of the heat source changes from the center of the FOV test image to the edge of the FOV test image, as shown in Figure 23b. The sensor in the left half can read the data of the heat source, whereas the sensor in the right half has no temperature data. At this time, the FOV of VSICE is the FOV of the sensor itself.

**Figure 24:**
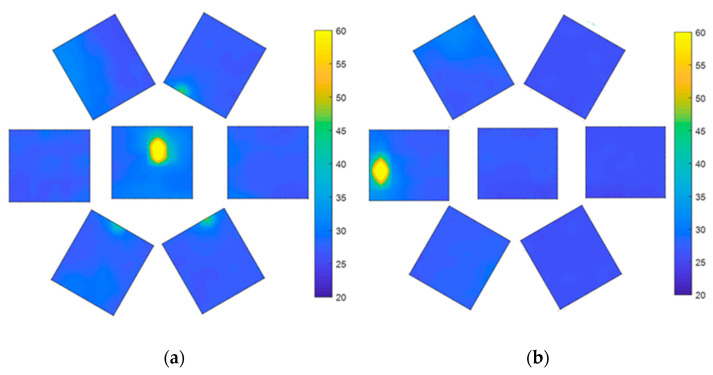
FOV test at 18 kPa. (**a**) Front test image of the compound eye. (**b**) Test image of the edge position of the compound eye rotation field.

When the air pressure is 18 kPa, the heat source can be detected by the central sensor (Figure 24), while the peripheral sensors have almost no complete heat source data. This is because the included angle φ between the FOV of the center sensor, and the side sensor becomes smaller after expansion. At this time, after the steering gear rotates by 60°, only the sensor at the left edge can measure the heat source. At this time, the FOV of the VSICE is twice the rotation angle of the steering gear, which increases the FOV compared with that at 0 Pa.

**Figure 25:**
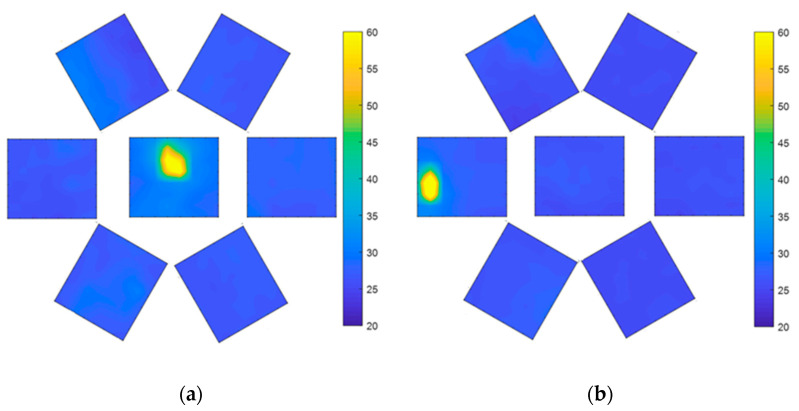
FOV test at 36 kPa. (**a**) Front test image of compound eye. (**b**) Test image of the compound eye rotated to the edge of the FOV.

When the air pressure is 36 kPa, the central sensor can detect the heat source (Figure 25a), while the marginal eyelet has no heat source data, which is also the reason for the incomplete 18 kPa heat source mentioned above. After the steering gear rotates by 70°, the left sensor can measure the heat source image, as shown in Figure 25b. At this time, the FOV of the VSICE is twice the rotation angle of the steering gear, which increases the FOV compared with that for the case of 18 kPa.

A comparison of Figure 23a, Figure 24a and Figure 25a shows that the heat source of the central eyelet sensor is amplified in the assembly, which is caused by the height change in the central eyelet of the compound eye during the expansion process.

The experimental results indicate that VSICE has a large enough angle range to adjust the FOV. By controlling the air pressure, VSICE can quickly convert a small FOV to a large FOV, stably read the array data under a large FOV, and can convert the planar compound eye to a curved compound eye. As the carrier is an SAM, the process of changing the FOV is reversible, which proves that SASCE can adapt to a variety of complex situations.

## 6. Discussion and Future Work

In this study, an array surface-changing compound eye based on soft pneumatics is proposed. The device has the characteristics of impact resistance, high stability, easy replacement, and strong applicability.

To verify the theoretical feasibility of SASCE, VSICE was established with SAM as the sensor carrier, and two simplified mathematical models are provided for readers to solve. Through FEA of CTPM by ABAQUS, the results of CTPM and SAM were compared, and the variation trends of its outer contour and air pressure, horizontal displacement and center distance under different air pressure, model thickness and air pressure were obtained. Then, CTPM and VSICE (with the same mathematical law of air-pressure driving as SAM) were photographed by filling gas in stages. The FEA results were compared with the outline measured in the experiment, the central points obtained from the FEA, experiment and mathematical model were compared with the changes in the air pressure point. The FEA results were compared with the side points measured in the experiment (attachment point or corresponding point of outer ring sensor) to verify the correctness of the FEA and mathematical model. The measured area of the sensor was obtained by analyzing the displacement points of the side points. An FOV measurement experiment of VSICE was carried out. From the FOV measurement experiment, the relationship between air pressure and FOV size was obtained, that is, the specified field angle of VSICE was obtained by controlling the air pressure.

Compared with the previous compound eye device, the proposed device has a stronger ability to adjust the FOV. Starting from adjusting the field angle, an SASCE is designed, which can expand the model by pumping gas, and stably change the array surface of the point compound eye formed by the infrared sensor, and the compound eye under different structures can stably return the temperature data and can process the digital image of these temperature data.

The proposed VSICE can pneumatically adjust the field angle. Driven by a 12 V mini-air pump, the VSICE can go from 0 Pa to 36 kPa in just 12 s. The speed of the VSICE’s FOV changes can be greatly improved by using a high-power air pump or by storing air pressure in advance. Due to its advantages in terms of adjustable range and reaction speed (determined by air-pressure differences), it can be further designed using other, more efficient sensors or cameras and by adopting arrays of different densities based on VSICE. When the load of a single sensor or camera is efficient enough and the load surface has enough elasticity, VSICE is expected to realize conversion from a planar compound eye to a nearly zero, dead angle, curved compound eye, which can be applied in the design of, and research on, artificial compound eye-tracking in the future.

The SASCE proposed in this paper has the advantage of a curved compound eye or planar compound eye in different states. Through VSICE, only the large FOV of SASCE surface state is studied, and changing the FOV is proved to affect the overlapping area. Compared with other, artificial eyes [20], the SASCE’s large size is a problem that must be solved. In the future, the research on SASCE’s overlapping field-of-view, optimization of the SASCE array, SASCE’s control algorithm, optimization of the SASCE model [21], and miniaturization of SASCE will be of great significance to the application of SASCE in high-speed tracking.

In this study, SASCE can quickly change the visual field and more efficiently use sensors. The change in the array surface of SASCE is nonlinear, and the change in the included angle position of the sensor is also nonlinear. To realize the tracking and seeking function, it also needs to cooperate with the steering gear pan tilt or mechanical arm. A combination of the SASCE and a mechanical arm can be controlled using adaptive neural control [22] generalized fuzzy neural adaptive control [23], adaptive fuzzy control [24], adaptive admittance control [25], adaptive neural control [26], and visual-fusion technology [27] to realize cooperative control of the compound eye array surface and rotary steering gear. Research on the control of a combination of SASCE and the manipulator is of great significance for the search for, and tracking, detection and operation [28] of, a specific target in the future.

## 7. Conclusions

In this study, an SASCE is proposed, which can change the array surface and adjust the FOV of a compound eye through pneumatic driving, to realize the driving of an SASCE from a planar state to curved state, so that it has the advantages of planar compound eye or curved compound eye in different states. SASCE can obtain more information about the overlapping FOV in the plane state, which can make the image formed by the overlapping FOV clearer or the spatial position of the apparent heat source more accurate. SASCE has a larger FOV in the curved state, which can make up for the loss of tracking targets caused by the slow response speed of the SASCE platform.

Through experiments on CTPM and VSICE, this paper proves the correctness of CTPM mathematical model, finite-element analysis and Sam finite-element analysis, as well as the possibility that SASCE can change FOV in a wide range, and the reality that SASCE changing the FOV will affect the size of overlapping field of view.

At the core of the changes in SASCE is software and pneumatic. The software factor makes the sensor more vulnerable to the vibrations of the impact point, and the pneumatic factor makes its working environment unable to contact sharp objects. However, compared with the existing compound eye, the compound eye in this paper can adjust a wider FOV.

## Data Availability

Not applicable.
